# Pathology and Treatment of Some of the Affections of the Upper Air Tract

**Published:** 1903-12

**Authors:** James M. Crawford

**Affiliations:** Atlanta, Ga.


### PATHOLOGY AND TREATMENT OF SOME OF THE AFFECTIONS OF THE UPPER AIR TRACT.*

By JAMES M. CRAWFORD, M.D.,

ATLANTA, GA.

It is safe to say that ninety per cent. of the affections in the upper air-passages, indiscriminately and indefinitely defined as "catarrh," are due to some form of obstruction, which may be bony or cartilaginous, or else hypertrophies of the softer parts in the nose or upper throat.

In this paper reference will be had to adenoids,—to the perverted vascular action in the nose and throat brought about by the exist-

*Read before the Georgia Medical Association, April, 1903.

ence of malformations, mainly of the septum, such as spurs, deflections, etc., and also to accute and chronic aural catarrh.

*Adenoids.*—Apropos of adenoids, too much honor can not be paid by our noble profession to the great Dane, Wilhelm Meyer, who in his able treatise written in 1869, gave to the medical world the method by which he determined positively the presence of a morbid growth in the vault of the pharynx, and here I wish to quote with approval the following tribute paid him by Dr. Jonathan Wright of New York:

"In seeking for the cause of an eustachian catarrh in patient, he pushed his finger above the velum palati, and thus became aware of a morbid growth, the removal of which has alleviated as much suffering and prevented as much disablement as any surgical procedure that was ever devised by the wit of man. Not only by his thorough exposé of the whole subject did Wilhelm Meyer thus confer an inestimable boon on suffering humanity, but he furnished a subsequent generation of rhinologists with their most lucrative source of income. No other event since the discovery of the laryngoscope has so contributed at once to the glory and the profit of the specialty of laryngology. May the modern rhinologist and his patient alike view with unstinted reverence the figure of Wilhelm Meyer as it stands in the Gefion Platz at Copenhagen."

Although this essay was written as you see in 1869 the profession here in the South seems to have paid but little attention to this branch of the subject until about 1889 and 1890—a period of twenty years—and then very timidly. It is interesting to compare the mode of treatment for such cases prior to 1889 and 1890 with that in vogue at the present day. At that time the methods employed were, I may truly say, empirical, being confined to certain set formulas for catarrh, etc., and though it is true attempts had been made for several years to examine the upper throat with the rhinoscope, very little had been accomplished and the post nares was still an unexplored part of anatomy. Within the last decade, however, great strides have been made, and the operation for the removal of adenoids is of almost daily occurrence.

*Structure.*—The structure of this hypertrophy of the adenoid tissue, known, variously as the "third tonsil," "Luschka's tonsil,"

and "adenoids," is so familiar to rhinologists of the present day that it would be superfluous for me to dwell upon it, and the symptoms so characteristic, that with the great light that has been shed upon the subject, diagnosis presents but little that is new. Indeed, were it not for its far-reaching and direful results, the whole subject might be considered too trite to bring before you.

*Diagnosis.*—Whenever physiognomy portrays a thin nose with nostrils collapsed and narrow, indicative of buccal inspiration; narrow jaws, the result usually of a high-arched palate; when complaint is made of difficult nocturnal breathing; of blood in the mouth or on the pillow in the morning; of a feeling of fullness in the upper throat behind the posterior nares, and when there is a chronic or frequent recurring attacks of accute coryza, the conclusion is obvious.

But however conclusive diagnosis by symptoms may be, I favor the direct method of a digital examination. The rhinoscope has in my experience proven to be so impracticable, the patient having, save in rare instances, to be educated to its use, I have almost abandoned it, proceeding at once to a digital examination, especially in the young where hypertrophied tonsils exist, and in many cases of eye troubles, since the lacrimal canals connect the mucous membrane of the lids with that of the nose and throat.

*Operative Procedure.*—Having found the adenoids, which may occur in any part of the laryngenial vault, the septum narium excepted, but which are more commonly found on the posterior and superior walls, I proceed to operate, using the curette, followed by the finger-nail for the smoothing and removal of any remaining hypertropied tissue.

Personally I do not favor the use of anesthetics, save that of cocain locally applied; but where, on account of extreme nervous irritability, a general anesthetic is desirable, I prefer chloroform, on account of the less disagreeable after-effects than those produced by ether, and because it can be more quickly administered. The operation is not a very painful one, being no worse than the removal of a pair of tonsils, and can be performed in about thirty seconds; therefore, I take issue with those who assert that it is cruel to remove adenoid growths, especially in children, without the use of a general anesthetic, and also claim that the physical shock is no greater than that produced by anesthesia.

When the patient is a child, I have my assistant to hold it in his lap, seated in an operating-chair. The child's hands are grasped by the assistant's right hand, while with his left he holds the patient's head firmly. Standing in front, and using a head mirror, I can readily see the patient's throat, and find no difficulty in quickly and safely removing the obstruction, using in most cases the Gottstein curette. So far I can report no casulties from hemorrhage.

*Treatment.*—After the operation, I prefer to treat the nose and throat daily, using a spray of Dobell's solution followed by an oil spray. This treatment I keep up more or less regularly until the parts are healed.

*Prognosis.*—When we consider that these obstructions in the nasopharyngeal vault not only exert a disturbing influence upon the adjacent cavities, but have been found to be a predisposing cause of some forms of kidney trouble, we can not lay too much stress upon the importance of their early removal. I do not wish to be understood as meaning that the existence of adenoids is the prime cause of acute or chronic Bright's, but that adenoids create a tendency to nephritis, and I am confident that where adenoids and nephritis occur simultaneously, that the removal of the former greatly increases the probability of a cure of the kidney trouble.

*Citation.*—Just here I wish to mention the case of a ten-year-old girl who had been suffering with acute aural catarrh from an existing adenoid. When, after a few days' treatment of the nose and throat with sprays, inflation of the eustachian tubes, etc., she was relieved of the acute symptoms, I appointed a time for the removal of the adenoid. The night preceding the appointed time I was called over the telephone by her family physician, Dr. W. S Elkin, and informed by him that the girl was suffering from acute Bright's; that he had found tube cast, hyaline cast and albumin, and that on that account he would deem it unwise to perform the operation. Consequently the operation was deferred for ten days, at the end of which time she was very much better, both from the cold and from the attack of nephritis. Unfortunately, however, she contracted another cold, and all of the other symptoms returned—albumin, tube and hyaline casts. When in a couple of weeks she had recovered in a measure from this second attack, the attending physician agreed to the operation, which I successfully performed in my office without an anesthetic. The physician reports to me that the symptoms of nephritis have disappeared entirely. This complication and happy termination has been observed by the writer on more occasions than the one here reported.

*Etiology of Adenoids.*—As to the etiology of adenoid vegetations, I am led to believe that they are largely hereditary. Nature is not so superficial as to give to a child the outer form of a parent's nose and ignore the underlying structures.

*Malformations of the Septum.*—Prominent among the obstructions causing perverted vascular action in the nose and throat are the several malformations of the septum, such as spurs, bends, deflections, etc. In many cases where we find the existence of adenoids we also find spurs or deflections, and this condition or complication I also ascribe, in a vast majority of cases, to heredity, and in but few to injuries.

*Surgery of the Septum.*—In the operative procedure for the removal of these bony or cartilaginous obstructions, we have made as great studies as in that for the removal of hypertrophies of the adenoid tissue. As there are no glands on the septum, it being simply a partition dividing the nostrils and acting as a framework, the malformations can readily be removed with the saw when skillfully and judiciously handled. By the use of a little 4 per cent. solution of muriate-cocain applied with a pledget of cotton, the pain from such an operation is reduced to a minimum.

*Legitimate use of Cocain.*—In this connection I wish to emphasize the point that *the use of muriate-cocain in nose and throat work should be that of relieving pain in cases of operation, and should not be used in the treatment following such operation.* I regret to say that we have cocain fiends being daily produced by such unprofessional methods.

*Treatment.*—The operation being accomplished, I pack the nose lightly with sterile gauze, removing the dressing at least once a day. A spray of Dobell's solution followed by an albolin spray should be used at each dressing. Too much cannot be said of the efficacy of Dobell's solution in cleansing the nose, and I fear we do not use it enough, depending too much on the albolin spray.

*Hemorrhage.*—Occasionally we meet with cases of profuse hemorrhage from this operation, but they seldom prove fatal, being quickly controlled by adrenalin, and by packing the nose with sterile, non-absorbent cotton. The discovery of adrenalin has been of vast importance, both for its admirable astringent properties and its value as a cardiac stimulant. In regard to instruments I have used various patterns of saws, but prefer the Bosworth.

*Acute and Chronic Aural Catarrh.*—These obstructions to which we have had reference are an important etiological factor in some of the diseases affecting the ear, and their removal is, I may say, the sheet-anchor in the treatment of most cases of acute or chronic aural catarrh. It is my usual custom to first search for these possible obstructions, and when their existence is discovered to immediately remove them.

*Inflation.*—I then endeavor to locate the ear trouble by the use of the tuning fork, and if found to be in the middle ear, inflation of the middle ear follows. In cases of internal nerve troubles inflation is of but little value. I seldom employ the eustachian catheter, but use instead compressed air from an air-receiver or tank at a pressure of about thirty-five pounds. I have found no trouble to arise from this method and prefer it to the catheter, inasmuch as it is more powerful and is more quickly manipulated.

As the eustachian tube is not only an air conductor to the middle ear, but is also a drainage-tube, you can readily see why I lay so much stress upon inflation or the opening of the natural outlet, the eustachian tube, especially when it is realized that when this method is employed perforation of the drum membrane is impossible.

If the satisfactory results of inflation in cases of acute aural catarrh were better known, there would be fewer advocates of the old-time prescription of laudanum and sweet oil, a perfectly useless procedure, since it does not reach the affected point. Every general practitioner should equip himself with a Politzer air-bag for use is cases of earache, as by it the tube which is closed by engorgement can be readily opened and relief quickly procured.

*Benefit to Posterity.*—To those of us who have seen a generation grow up since we entered upon our practice, and have seen the development that a score of years has made in our profession, especially in Georgia, the pleasing thought arises that the coming generation will be the greatest beneficiaries of this skill and research.

Not only will they inherit a more vigorous physique from those who have had the benefit of this skill, but along with that physique will come a more active mentality; and in the treatment of such disorders as may attack them they will have the benefit of a highly trained and studious profession. Adding to this increased professional knowledge the better education of our fellow citizens, let us hope that the quack and his nostrums will vanish from the earth.

